# Nationwide survey of the development of drug-resistant pathogens in the pediatric field in 2007 and 2010: drug sensitivity of *Streptococcus pneumoniae* in Japan (second report)

**DOI:** 10.1007/s10156-013-0593-x

**Published:** 2013-04-07

**Authors:** Takeshi Tajima, Yoshitake Sato, Yoshikiyo Toyonaga, Hideaki Hanaki, Keisuke Sunakawa

**Affiliations:** 1 Department of Pediatrics, Hakujikai Memorial Hospital, 5-11-1 Shikahama, Adachi-ku, Tokyo, Japan; 2 Department of Pediatrics, Fuji Heavy Industries Health Insurance Society, Ota Memorial Hospital, Ota, Japan; 3 Department of Pediatrics, Sekishinkai Sayama Hospital, Sayama, Japan; 4 The Kitasato Institute, Kitasato University Research Center for Anti-infectious Drugs, Tokyo, Japan; 5 The Kitasato Institute, Kitasato University Research Organization for Infection Control Sciences, Tokyo, Japan

**Keywords:** Drug resistance, Pediatric infectious disease, Sensitivity, *Streptococcus pneumoniae*, Surveillance

## Abstract

We previously conducted nationwide surveillance of *Streptococcus pneumoniae* in 2000–2001 (period 1) and 2004 (period 2) and reported the findings. Subsequent surveillance surveys conducted in 2007 (period 3) and 2010 (period 4) are now reported. Bacterial strains were clinically isolated from children with meningitis, sepsis, and respiratory tract infections at 27 hospitals participating in the Drug-Resistant Pathogen Surveillance Group in Pediatric Infectious Disease. Twenty-one drugs were investigated for 283 isolated strains in period 3, and 24 drugs were investigated for 459 strains in period 4. In period 3, 43.8 % of strains were penicillin-susceptible *S. pneumoniae* (PSSP), 52.3 % were penicillin-intermediate *S. pneumoniae* (PISP), and 3.9 % were penicillin-resistant *S. pneumoniae* (PRSP). In period 4, the percentages were PSSP 23.1 %, PISP 49.9 %, and PRSP 27.0 %. The resistance rates were 56.2 % and 76.9 %, respectively. Drug sensitivity was best with panipenem, at a minimum inhibitory concentration (MIC)_90_ ≤0.063 μg/ml in period 3, and with tebipenem (MIC_90_ ≤ 0.063 μg/ml) in period 4. Patients’ background factors related to increased bacterial resistance were investigated, and significant differences were found depending on whether a child had siblings (*P* = 0.0056) or was a daycare center attendee (*P* = 0.0195) in period 3, and age category (*P* = 0.0256) in period 4. No factors were common to both periods 3 and 4. *Pneumococcus* is a major causative organism of pediatric infectious disease, and we plan to continue conducting surveillance and providing information in the future.

## Introduction

Together with *Haemophilus influenzae* and *Moraxella catarrhalis*, *Streptococcus pneumoniae* is a main causative organism of otorhinolaryngological infections and respiratory tract infections in children. *S. pneumoniae* in particular is a major causative organism for sepsis and purulent meningitis after infancy, and it is considered to be one of the most important bacterial strains causing infections [1, 2].

In recent years, the increase in penicillin-intermediate *S. pneumoniae* (PISP) and penicillin-resistant *S. pneumoniae* (PRSP), which show resistance to penicillins, is becoming a problem. It has also been reported that sensitivity to cephems and carbapenems is decreasing, which is a factor in making respiratory tract and otorhinolaryngological infections in children more intractable and their treatment more difficult [3].

Amid the trend of increasing resistance of *S. pneumoniae*, our group has surveyed drug sensitivity over time since 2000. The results of nationwide surveys conducted in 2007 (period 3) and 2010 (period 4), following earlier surveys in 2000–2001 (period 1) and 2004 (period 2) [4], are now reported.

## Materials and methods

### Subjects

Among the strains clinically isolated from children with meningitis, sepsis, and respiratory infection at 27 facilities that participated in the nationwide Drug-Resistant Pathogen Surveillance Group in Pediatric Infectious Disease between January and June 2007 (period 3) and January and June 2010 (period 4), 283 strains and 459 strains were investigated in each period, respectively, that were judged by the physicians in charge to be causative organisms based on the amount of bacteria, laboratory test, and patients’ clinical symptoms.

The number of strains isolated from various specimens was calculated in each period, and the following results were obtained: blood (7 strains), swabs collected from the nasopharynx (251 strains), sputum (21 strains), ear discharge (1 strain), and unknown (3 strains) in period 3, and blood (23 strains), spinal fluid (1 strain), swabs collected from the nasopharynx (392 strains), sputum (41 strains), and unknown (2 strains) in period 4.

In addition, to determine the origin of each strain, factors such as sex, age, presence or absence of siblings, presence or absence of group childcare, presence or absence of previous treatment with antibacterial agents, and the location of the hospital were investigated. The present study was reviewed and approved by the ethics committee of each facility in accordance with their regulations.

### Measured drugs

In testing sensitivity, 21 drugs were used in period 3 [penicillin G (PCG), ampicillin (ABPC), amoxicillin (AMPC), cefaclor (CCL), cefditoren (CDTR), cefcapene (CFPN), cefpodoxime (CPDX), cefdinir (CFDN), cefotiam (CTM), ceftriaxone (CTRX), cefotaxime (CTX), cefteram (CFTM), azithromycin (AZM), clarithromycin (CAM), rokitamycin (RKM), faropenem (FRPM), panipenem (PAPM), meropenem (MEPM), vancomycin (VCM), telithromycin (TEL), and levofloxacin (LVFX)]; and 24 drugs were used in period 4 [PCG, ABPC, AMPC, piperacillin (PIPC), CCL, CDTR, CFPN, CPDX, CFDN, CTM, CTRX, CTX, CFTM, AZM, CAM, RKM, FRPM, PAPM, MEPM, doripenem (DRPM), tebipenem (TBPM), VCM, LVFX, and tosufloxacin (TFLX)].

### Sensitivity testing and analysis

Isolation, culturing, and identification were conducted at each facility, and all sensitivity measurements were conducted at Kitasato University. To test sensitivity, the minimum inhibitory concentration (MIC) was measured using the broth microdilution method [5] according to Clinical and Laboratory Standards Institute (CLSI) methods. Then, in accordance with the CLSI standards for oral antibacterial agents (nonmeningitis1, oral penicillin), PCG sensitivity of ≤0.06 μg/ml was classified as PSSP, sensitivity of 0.12–1.0 μg/ml was classified as PISP, and sensitivity of ≥2.0 μg/ml was classified as PRSP [6].

In addition, strains from sterile sites accounted for less than 10 % of the overall strains, specifically 7 strains (blood) in period 3 and 24 strains in period 4 (cerebrospinal fluid, *n* = 1; blood, *n* = 23), and therefore no investigation by origin was conducted for them.

An analysis by patient background was also done to identify factors related to increased resistance. The analysis was done using the* χ* ^2^ test with a two-sided significance level of 0.05. In cases when the expected value was ≤10, Fisher’s exact probability test was used.

## Results

### Drug sensitivity

The drug sensitivity results are shown in Table 1 for period 3 (2007) and in Table 2 for period 4 (2010).

**Table 1:** Drug susceptibility in period 3 (2007)

Drug	MIC range (μg/ml)	*S. pneumoniae*		PSSP		PISP		PRSP	
		MIC_50_ (μg/ml)	MIC_90_ (μg/ml)	MIC_50_ (μg/ml)	MIC_90_ (μg/ml)	MIC_50_ (μg/ml)	MIC_90_ (μg/ml)	MIC_50_ (μg/ml)	MIC_90_ (μg/ml)
PCG	≤0.063–2	0.25	1	≤0.063	≤0.063	0.5	1	2	2
ABPC	≤0.063–8	0.25	2	≤0.063	≤0.063	1	2	4	4
AMPC	≤0.063–4	0.25	1	≤0.063	≤0.063	0.5	1	1	4
CCL	0.125–128	4	64	0.5	4	16	64	128	128
CDTR	≤0.063–4	0.125	0.25	≤0.063	0.25	0.25	0.5	0.5	0.5
CFPN	≤0.063–8	0.25	0.5	0.125	0.5	0.5	1	1	1
CPDX	≤0.063–64	1	2	0.25	1	1	2	2	4
CFDN	≤0.063–16	1	4	0.25	1	2	4	4	8
CTM	≤0.063–8	1	4	0.25	1	2	4	4	8
CTRX	≤0.063–8	0.25	1	0.125	0.5	0.5	1	1	1
CTX	≤0.063–8	0.5	0.5	0.125	0.5	0.5	1	1	1
CFTM	≤0.063–8	0.25	1	0.125	0.5	0.5	1	1	1
AZM	≤0.063–64	>64	>64	>64	>64	>64	>64	>64	>64
CAM	≤0.063 to >64	>64	>64	64	>64	>64	>64	>64	>64
RKM	≤0.063 to >32	1	>32	2	>32	1	>32	>32	>32
FRPM	≤0.063–1	≤0.063	0.25	≤0.063	≤0.063	0.25	0.25	0.5	0.5
PAPM	≤0.063–0.5	≤0.063	≤0.063	≤0.063	≤0.063	≤0.063	0.125	≤0.063	0.25
MEPM	≤0.063–0.5	≤0.063	0.25	≤0.063	≤0.063	0.125	0.25	0.5	0.5
VCM	0.125–0.5	0.25	0.25	0.25	0.25	0.25	0.25	0.25	0.25
TEL	≤0.063–1	≤0.063	0.25	≤0.063	0.25	≤0.063	0.25	0.125	0.5
LVFX	0.5–2	1	1	1	2	1	1	1	2

*ABPC* ampicillin, *AMPC* amoxicillin, *AZM* azithromycin, *CAM* clarithromycin, *CCL* cefaclor, *CDTR* cefditoren, *CFDN* cefdinir, *CFTM* cefteram, *CPDX* cefpodoxime, *CTM* cefotiam, *CTRX* ceftriaxone, *CTX* cefotaxime, *FRPM* faropenem, *LVFX* levofloxacin, *MEPM* meropenem, *PAPM* panipenem, *PCG* penicillin G, *PISP* penicillin-intermediate *S. pneumoniae*, *PRSP* penicillin-resistant *S. pneumoniae*, *PSSP* penicillin-susceptible *S. pneumoniae*, *RKM* rokitamycin, *TEL* telithromycin, *VCM* vancomycin

**Table 2:** Drug susceptibility in period 4 (2010)

Drug	MIC range (μg/ml)	*S. pneumoniae*		PSSP		PISP		PRSP	
		MIC_50_ (μg/ml)	MIC_90_ (μg/ml)	MIC_50_ (μg/ml)	MIC_90_ (μg/ml)	MIC_50_ (μg/ml)	MIC_90_ (μg/ml)	MIC_50_ (μg/ml)	MIC_90_ (μg/ml)
PCG	≤0.063–4	1	2	≤0.063	≤0.063	0.5	1	2	2
ABPC	≤0.063–8	0.5	2	≤0.063	0.125	0.5	2	2	4
AMPC	≤0.063–4	0.25	1	≤0.063	≤0.063	0.25	1	1	2
PIPC	≤0.063–4	0.5	2	≤0.063	≤0.063	0.5	2	2	2
CCL	≤0.063–128	16	64	1	2	16	32	64	128
CDTR	≤0.063–8	0.25	1	0.125	0.5	0.25	0.5	0.5	1
CFPN	≤0.063–16	0.5	1	0.25	0.5	0.5	1	1	2
CPDX	≤0.063–16	1	2	0.5	2	1	2	2	4
CFDN	≤0.063–32	2	8	0.25	1	2	4	4	8
CTM	≤0.063–16	1	8	0.25	0.5	1	4	4	8
CTRX	≤0.063–8	0.5	1	0.25	0.5	0.5	1	1	2
CTX	≤0.063–16	0.5	1	0.25	0.5	0.5	1	1	2
CFTM	≤0.063–16	0.5	1	0.25	1	0.5	1	1	2
AZM	≤0.063 to >64	>64	>64	>64	>64	>64	>64	>64	>64
CAM	≤0.063 to >64	>64	>64	>64	>64	>64	>64	>64	>64
RKM	≤0.063–>32	2	>32	8	>32	2	>32	2	>32
FRPM	≤0.063–1	0.125	0.5	≤0.063	≤0.063	0.125	0.25	0.25	0.5
PAPM	≤0.063–0.5	≤0.063	0.125	≤0.063	≤0.063	≤0.063	0.125	≤0.063	0.25
MEPM	≤0.063–1	0.125	0.5	≤0.063	≤0.063	0.125	0.25	0.25	0.5
DRPM	≤0.063–1	≤0.063	0.5	≤0.063	≤0.063	≤0.063	0.25	0.25	0.5
TBPM	≤0.063–0.125	≤0.063	≤0.063	≤0.063	≤0.063	≤0.063	≤0.063	≤0.063	≤0.063
VCM	≤0.063–0.5	0.25	0.5	0.25	0.5	0.25	0.5	0.25	0.5
LVFX	0.5–2	1	2	1	2	1	2	1	2
TFLX	≤0.063–0.5	0.25	0.25	0.25	0.25	0.25	0.25	0.25	0.25

*ABPC* ampicillin, *AMPC* amoxicillin, *AZM* azithromycin, *CAM* clarithromycin, *CCL* cefaclor, *CDTR* cefditoren, *CFDN* cefdinir, *CFTM* cefteram, *CPDX* cefpodoxime, *CTM* cefotiam, *CTRX* ceftriaxone, *CTX* cefotaxime, *FRPM* faropenem, *LVFX* levofloxacin, *MEPM* meropenem, *PAPM* panipenem, *PCG* penicillin G, *PIPC* piperacillin*, PISP* penicillin-intermediate *S. pneumoniae*, *PRSP* penicillin-resistant *S. pneumoniae*, *PSSP* penicillin-susceptible *S. pneumoniae*, *RKM* rokitamycin, *TEL* telithromycin, *VCM* vancomycin

In period 3, 124 (43.8 %) strains were PSSP, 148 (52.3 %) strains were PISP, and 11 (3.9 %) strains were PRSP. PISP + PRSP accounted for 56.2 %. The best MIC_90_ of the antibacterial agents tested was for PAPM (≤0.063 μg/ml), followed by CDTR, FRPM, MEPM VCM, and TEL (0.25 μg/ml). By type of sensitivity, the MIC_90_ values of PCG, ABPC, AMPC, FRPM, PAPM, and MEPM were the most active for PSSP, at ≤0.063 μg/ml. PAPM had an MIC_90_ value of 0.125 μg/ml for PISP, and PAPM and VCM had MIC_90_ values of 0.25 μg/ml for PRSP.

In period 4, 106 (23.1 %) strains were PSSP, 229 (49.9 %) strains were PISP, and 124 (27.0 %) strains were PRSP. PISP + PRSP accounted for 76.9 %. The best MIC_90_ of the antibacterial agents tested was for TBPM (≤0.063 μg/ml), followed by PAPM (0.125 μg/ml). With respect to the MIC_90_ for PSSP, the best antibacterial agents were PCG, AMPC, PIPC, FRPM, TBPM, PAPM, MEPM, and DRPM, at ≤0.063 μg/ml. TBPM had the best MIC_90_ values for PISP and PRSP, at ≤0.063 μg/ml.

### Resistance rates for each background factor

The resistance rates for background factors for the strains and the resistance rate with each background factor in periods 3 and 4 are shown in Tables 3 and 4, respectively.

**Table 3:** Resistance rate for each background factor in period 3 (2007)

Background factor	*S. pneumoniae*	PSSP	PISP	PRSP	Resistant rate	Statistical test
	Number of strains	Number of strains (%)	Number of strains (%)	Number of strains (%)	(%)	
Total	283	124 (43.8)	148 (52.3)	11 (3.9)	56.2	
Sex						
Male	148	66 (44.6)	78 (52.7)	4 (2.7)	55.4	Fisher
Female	124	53 (42.7)	64 (51.6)	7 (5.6)	57.3	*P* = 0.9537
Unknown	11	5 (45.5)	6 (54.5)	0 (0.0)	54.5	
Age category						
Infant	57	25 (43.9)	30 (52.6)	2 (3.5)	56.1	Fisher
Toddler and preschooler	203	84 (41.4)	110 (54.2)	9 (4.4)	58.6	*P* = 0.0663
School child	22	15 (68.2)	7 (31.8)	0 (0.0)	31.8	
Unknown	1	0 (0.0)	1 (100.0)	0 (0.0)	100.0	
Siblings						
No	129	45 (34.9)	76 (58.9)	8 (6.2)	65.1	Fisher
Yes	153	79 (51.6)	71 (46.4)	3 (2.0)	48.4	*P* = 0.0056
Unknown	1	0 (0.0)	1 (100.0)	0 (0.0)	100.0	
Daycare center attendee						
No	111	51 (45.9)	58 (52.3)	2 (1.8)	54.1	Fisher
Yes	155	71 (45.8)	75 (48.4)	9 (5.8)	54.2	*P* = 0.0195
Unknown	17	2 (11.8)	15 (88.2)	0 (0.0)	88.2	
Daycare center attendee (siblings)						
No	25	12 (48.0)	12 (48.0)	1 (4.0)	52.0	*χ* ^2^
Yes	116	63 (54.3)	51 (44.0)	2 (1.7)	45.7	*P* = 0.5663
Unknown	142	49 (34.5)	85 (59.9)	8 (5.6)	65.5	
Previous antibacterial agents						
No	125	61 (48.8)	58 (46.4)	6 (4.8)	51.2	Fisher
Yes	156	62 (39.7)	89 (57.1)	5 (3.2)	60.3	*P* = 0.2488
Unknown	2	1 (50.0)	1 (50.0)	0 (0.0)	50.0	
Penicillins						
Yes	29	5 (17.2)	21 (72.4)	3 (10.3)	82.8	
Unknown	254	119 (46.9)	127 (50.0)	8 (3.1)	53.1	
Cephems						
Yes	100	40 (40.0)	57 (57.0)	3 (3.0)	60.0	
Unknown	183	84 (45.9)	91 (49.7)	8 (4.4)	54.1	
Macrolides						
Yes	53	28 (52.8)	23 (43.4)	2 (3.8)	47.2	
Unknown	230	96 (41.7)	125 (54.3)	9 (3.9)	58.3	

*PISP* penicillin-intermediate *S. pneumoniae*, *PRSP* penicillin-resistant *S. pneumoniae*, *PSSP* penicillin-susceptible *S. pneumoniae*

**Table 4:** Resistance rate for each background factor in period 4 (2010)

Background factor	*S. pneumoniae*	PSSP	PISP	PRSP	Resistant rate	Statistical test
	Number of strains	Number of strains (%)	Number of strains (%)	Number of strains (%)	(%)	
Total	459	106 (23.1)	229 (49.9)	124 (27.0)	76.9	
Sex						
Male	272	65 (23.9)	133 (48.9)	74 (27.2)	76.1	Fisher
Female	186	41 (22.0)	95 (51.1)	50 (26.9)	78.0	*P* = 0.7339
Unknown	1	0 (0.0)	1 (100.0)	0 (0.0)	100.0	
Age category						
Infant	98	21 (21.4)	51 (52.0)	26 (26.5)	78.6	*χ* ^2^
Toddler and preschooler	330	71 (21.5)	164 (49.7)	95 (28.8)	78.5	*P* = 0.0256
Schoolchild	31	14 (45.2)	14 (45.2)	3 (9.7)	54.8	
Siblings						
No	185	35 (18.9)	96 (51.9)	54 (29.2)	81.1	*χ* ^2^
Yes	274	71 (25.9)	133 (48.5)	70 (25.5)	74.1	*P* = 0.0812
Daycare center attendee						
No	180	44 (24.4)	91 (50.6)	45 (25.0)	75.6	Fisher
Yes	251	55 (21.9)	126 (50.2)	70 (27.9)	78.1	*P* = 0.7893
Unknown	28	7 (25.0)	12 (42.9)	9 (32.1)	75.0	
Daycare center attendee (siblings)						
No	45	10 (22.2)	26 (57.8)	9 (20.0)	77.8	Fisher
Yes	206	56 (27.2)	98 (47.6)	52 (25.2)	72.8	*P* = 0.1586
Unknown	208	40 (19.2)	105 (50.5)	63 (30.3)	80.8	
Previous antibacterial agents						
No	204	51 (25.0)	103 (50.5)	50 (24.5)	75.0	*χ* ^2^
Yes	255	55 (21.6)	126 (49.4)	74 (29.0)	78.4	*P* = 0.3861
Penicillins						
Yes	60	13 (21.7)	32 (53.3)	15 (25.0)	78.3	
Unknown	399	93 (23.3)	197 (49.4)	109 (27.3)	76.7	
Cephems						
Yes	127	29 (22.8)	67 (52.8)	31 (24.4)	77.2	
Unknown	332	77 (23.2)	162 (48.8)	93 (28.0)	76.8	
Macrolides						
Yes	107	21 (19.6)	50 (46.7)	36 (33.6)	80.4	
Unknown	352	85 (24.1)	179 (50.9)	88 (25.0)	75.9	

*PISP* penicillin-intermediate *S. pneumoniae*, *PRSP* penicillin-resistant *S. pneumoniae*, *PSSP* penicillin-susceptible *S. pneumoniae*

Looking at the background factors for 283 strains in period 3, ≥70 % of strains were from toddlers and preschoolers (71.7 %, 203 strains). Other background factors were being male (52.3 %, 148 strains), having siblings (54.1 %, 153 strains), daycare center attendee (54.8 %, 155 strains), and having previously received antibacterial agents (55.1 %, 156 strains). No significant differences were seen.

Of the background factors for 459 strains in period 4, the most were related to being male (59.3 %, 272 strains), being a toddler or preschooler (71.9 %, 330 strains), and having siblings (59.7 %, 274 strains). Other background factors were daycare center attendee (54.7 %, 251 strains) and having previously received antibacterial agents (55.6 %, 255 strains). However, no significant differences were seen.

In period 3, drug-resistant strains were related to daycare center attendance (*P* = 0.0195). In period 4, there were significantly more such strains by age category (*P* = 0.0256).

## Discussion

Because penicillins have shown superior antibacterial activity against *S. pneumoniae*, refractory cases of pediatric respiratory tract infections have been rare. In recent years, however, PRSP that show resistance to penicillins, cephems, and carbapenems are increasing rapidly. The number of cases in which there is difficulty in treating acute otitis media or respiratory tract infections is growing, and the increasing drug resistance of *S. pneumoniae* is becoming a serious problem. [4].

Surveillance of *S. pneumoniae* strains derived from pediatric infections was conducted to confirm the state of drug resistance increases in *S. pneumoniae* from October 2000 to July 2001 (period 1) and from January to June 2004 (period 2), and the findings were reported. Subsequent surveillance surveys were then conducted in period 3 and period 4.

The resistance rates were 56.2 % in period 3 and 76.9 % in period 4, more than half in both periods and showing an increasing trend. A rapid increase was seen in PRSP in particular, from 3.9 to 27.0 %. Investigation of the resistance rate by background factor revealed no common factors that showed significant differences in both period 3 and period 4, despite the significant differences seen with age category in periods 4 and 3, respectively. At the current time, no individual factor responsible for the increases in bacterial resistance can be identified.

However, PISP + PRSP accounted for 64.6 % in period 1, 66.9 % in period 2, 56.2 % in period 3, and 76.9 % in period 4, with a decrease in period 3. Looking at PRSP only, the proportions were 29.8, 29.8, 3.9, and 27.0 %, respectively, decreasing markedly in period 3.

Jaecklin et al. conducted a survey on patients under 16 years old in Switzerland [7] and reported the isolation frequency of PISP + PRSP between 1989 and 2004. The isolation frequency, which was 2 % in 1989, maintained a 20 % level during the period after 1994 and reached 41 % in 2004. The trend observed in this survey was similar to that observed in the Japanese survey, although these surveys were conducted during different periods. Because pneumococci are becoming increasingly resistant to drugs around the world, close surveillance should be conducted continuously.

In a nationwide survey conducted by Suzuki et al. [8] in the field of otorhinolaryngology, the isolation frequencies in 1994, 1998, 2003, and 2008 were 36.1, 29.1, 39.7, and 33.3 %, respectively, for PISP, 14.3, 21.8, 19.9, and 12.8 %, respectively, for PRSP, and 50.4, 50.9, 59.6, and 46.1 %, respectively, for PISP + PRSP. In 2008, marked decreases were seen in the proportions of PRSP and PISP + PRSP. Although there was a 1-year discrepancy in the years in which the surveys were conducted, these trends were similar to the present findings.

Clinical practice guidelines have been published one after another, including the “Guidelines for the Management of Respiratory Infectious Diseases in Children in Japan” [9] in 2004, the “JRS Guidelines for the Management of Community-Acquired Pneumonia in Adults” [10] in 2005, and the “Treatment Guidelines for Acute Otitis Media in Children” [11]. The Guidelines for the Management of Respiratory Infectious Diseases in Children in Japan were revised in 2007 and 2011, and the Treatment Guidelines for Acute Otitis Media in Children were revised in 2009 for the edification of doctors and to encourage proper use of antibacterial agents, including new drugs. In addition, a *S. pneumoniae* vaccine for children put on the market in 2010 has been gradually changing the treatment of pediatric infections.

The results in this report may have been affected by the above-described change in clinical practice, and more detailed investigations are needed, for example, by drug use.

With regard to antibacterial sensitivity, drugs with an MIC_90_ ≤ 1 μg/ml for PRSP in period 3 and/or period 4 were CDTR, CFPN, CTRX, CTM, CFTM, FRPM, TBPM, PAPM, MEPM, DRPM, VCM, TEL, and TFLX. After excluding DRPM, TBPM, and TFLX, which were only measured in period 4 and TEL, which was only measured in period 3, only MEPM of the nine remaining drugs showed a greater than fourfold increase in MIC _90_ from period 1 to period 4. *S. pneumoniae* is one of the major etiological agents in pediatric infections, and based on the sensitivity shown in this study, it is thought to be necessary to select antibacterial agents based on drug disposition and the administration route.

Ascertaining resistance status will lead not only to recommendations for appropriate antimicrobial treatment, but also to controlling drug-resistant pathogens, and we plan to continue conducting surveillance and providing information in the future.
